# Six Decades of Methodological Evolution in Portuguese Food Balance Sheets: Implications for Nutrition Surveillance and Food System Monitoring

**DOI:** 10.3390/nu18142341

**Published:** 2026-07-16

**Authors:** Beatriz Santos, Alexandra Bento, Leandro Oliveira, Pedro Nabais, António Raposo, Maria do Céu Costa

**Affiliations:** 1 ECTS (School of Health Sciences and Technologies), Lusófona University, Campo Grande 376, 1749-024 Lisboa, Portugal; beatriz.santos2001@hotmail.com; 2 Food and Nutrition Department, National Health Institute Doutor Ricardo Jorge, Av. Padre Cruz, 1649-016 Lisboa, Portugal; alexandra.bento@insa.min-saude.pt; 3 CBIOS (Research Center for Biosciences and Health Technologies), ECTS (School of Health Sciences and Technologies), Lusófona University, Campo Grande 376, 1749-024 Lisboa, Portugal; leandro.oliveira@ulusofona.pt (L.O.); maria.costa@ulusofona.pt (M.d.C.C.); 4 Food Risks Unit, Economic and Food Safety Authority (ASAE), 1649-038 Lisboa, Portugal; pmnabais@asae.pt; 5 Centro de Investigação do IPLuso (CIPLuso), Polytechnic Institute of Lusophony, ERISA—Escola Superior de Saúde Ribeiro Sanches, Rua do Telhal aos Olivais, 8, 1950-396 Lisboa, Portugal

**Keywords:** food availability, Food Balance Sheets, food system monitoring, methodological evolution, nutrition surveillance, Supply and Utilization Accounts

## Abstract

**Background:** Food Balance Sheets (FBS) are widely used to assess national food availability, monitor nutrition transitions, and support food and nutrition policies. However, methodological revisions may influence the comparability of food availability estimates and nutrient supply indicators over time. This study aimed to examine the methodological evolution of Portuguese FBS between 1963 and 2024 and to assess its implications for nutrition surveillance and food system monitoring. **Methods:** A retrospective documentary analysis was conducted using all official Portuguese FBS published by the National Statistics Institute between 1963 and 2024. Methodological characteristics were systematically compared across six reporting periods, including institutional frameworks, data sources, food classification systems, nutritional indicators, food availability equations, food loss estimation procedures, and nutrient conversion methods. **Results:** Substantial methodological developments were identified over the six-decade period. Portuguese FBS evolved from relatively simple food supply accounting systems towards increasingly standardized frameworks aligned with the Food and Agriculture Organization Supply and Utilization Accounts methodology. Major changes included the incorporation of food losses, animal feed, industrial uses, and seed utilization into food availability calculations; the expansion of reported nutritional indicators from energy, protein, and fat to include carbohydrates, vitamins, and minerals; and the progressive refinement of food classification systems. More recent editions also introduced additional nutritional indicators, enhancing the characterization of food availability and nutrient supply. These developments may have contributed to expanding the nutritional scope of Portuguese FBS, strengthening methodological standardization, and improving alignment with international standards. **Conclusions:** Over the last six decades, Portuguese FBS have undergone substantial methodological modernization and increasing alignment with international standards. Although FBS remain indirect measures of food consumption, their evolution has strengthened their value for population-level food availability analyses, nutrition surveillance, food system monitoring, and evidence-informed policymaking. The findings also highlight the importance of considering methodological changes when interpreting long-term trends in food availability and nutrient supply.

## 1. Introduction

Food systems play a fundamental role in shaping population health, food security, environmental sustainability, and economic development. Understanding how food availability changes over time is therefore essential for monitoring food supply trends, evaluating nutrition transitions, and informing evidence-based nutrition and public health policies [1,2,3]. In this context, Food Balance Sheets (FBS) have become one of the most widely used tools for assessing national food supplies and monitoring long-term changes in food availability at the population level [4,5,6,7].

FBS provide a comprehensive overview of national food supply by integrating data on agricultural production, imports, exports, stock variations, industrial utilization, animal feed, seed use, and food losses [4,5]. The resulting estimates are generally expressed as per capita daily food availability and nutrient supply, including energy, protein, fat, carbohydrates, vitamins, and minerals [8,9]. Although FBS do not directly measure food consumption, they provide valuable information on the nutritional capacity of national food systems and constitute an important source of data for nutrition surveillance, food security assessments, and analyses of long-term food availability trends [6,7,10].

The scientific relevance of FBS has expanded considerably over recent decades. Initially developed as agricultural and food security monitoring tools, FBS are now increasingly used to investigate nutrition transitions, evaluate changes in national food availability, assess food system sustainability, and examine the relationship between food availability and health outcomes [6,10,11]. Previous studies have shown that changes in national food supplies frequently coincide with increased availability of animal-source foods, added sugars, vegetable oils, and ultra-processed foods. These changes in food availability have been associated with the growing burden of obesity, cardiovascular diseases, type 2 diabetes, and other non-communicable diseases worldwide [11,12,13,14].

FBS are also increasingly recognized as valuable tools for assessing food system sustainability. Contemporary food systems account for approximately one-third of global anthropogenic greenhouse gas emissions and contribute substantially to land degradation, freshwater depletion, and biodiversity loss [1,2]. Consequently, researchers and policymakers are increasingly using FBS data to evaluate the environmental implications of national food availability, assess the alignment between food supplies and dietary recommendations, and monitor progress towards healthier and more sustainable food systems [15,16,17,18,19]. Such applications are particularly relevant within the framework of the Sustainable Development Goals and the growing international emphasis on sustainable diets capable of simultaneously promoting human and planetary health [1,18,19].

Within the Mediterranean region, FBS have been widely used to investigate long-term changes in national food availability and to evaluate the extent to which national food supplies align with Mediterranean dietary recommendations. Several studies have documented substantial changes in food availability across Mediterranean countries, including increased availability of animal products, fats, sugars, and processed foods, indicating a progressive shift away from traditional Mediterranean dietary characteristics [20,21,22]. These findings are particularly relevant because the Mediterranean diet is widely recognized as one of the healthiest and most sustainable dietary models, providing benefits for human health, environmental sustainability, cultural heritage preservation, and economic resilience [18,23,24].

Portugal has experienced similar changes in national food availability over recent decades. Analyses based on Portuguese FBS data have documented significant increases in the availability of animal-source foods, fats, and energy-dense products, reflecting broader nutrition transition processes observed across Southern Europe [21,25,26]. More recently, studies have highlighted growing concerns regarding the increasing availability of ultra-processed foods and their potential implications for population health [14]. Furthermore, evidence from Portuguese populations indicates that adherence to Mediterranean dietary recommendations remains suboptimal in several demographic groups, particularly among younger adults, emphasizing the importance of robust food monitoring systems capable of tracking changes in national food availability and food supply patterns over time [27].

The development of Portuguese FBS is closely linked to the historical evolution of nutrition and food policy in Portugal. During the Second World War and the immediate post-war period, concerns regarding food shortages, undernutrition, and poor nutritional conditions prompted efforts to improve understanding of the nutritional status of the Portuguese population [28]. Several pioneering investigations conducted during this period identified important deficiencies in energy and animal protein availability and highlighted significant challenges related to maternal and child nutrition [28].

In response to these challenges, the Portuguese government established a formal collaboration with the Food and Agriculture Organization of the United Nations (FAO) in 1951 to support nutrition research and food system monitoring [28]. As part of this initiative, the Portuguese National Statistics Institute was assigned responsibility for developing the first Portuguese FBS, creating one of the earliest national systems for monitoring food availability in Europe [5,28].

The Portuguese FBS system currently represents one of the longest-running food monitoring programmes in Europe. Official FBS datasets are available for the periods 1963–1975 [28], 1980–1992 [29], 1990–1997 [30], 2012–2016 [31], 2016–2020 [8], and 2020–2024 [32]. Throughout this period, substantial methodological developments have occurred, reflecting advances in nutritional science, improvements in statistical systems, increasing data availability, and successive revisions of FAO methodological guidelines [4,7]. Particularly important was the adoption of the Supply and Utilization Accounts (SUA) framework, which introduced significant modifications in food availability calculations, food loss estimation, industrial utilization accounting, livestock feed allocation, and nutrient supply assessment [4,10].

Although FBS are widely used to examine long-term changes in national food availability and to support research on nutrition transitions and food system transformations, the validity of longitudinal analyses depends on the methodological consistency of the underlying data. Revisions in food classification systems, nutrient reporting procedures, food loss estimation methods, conversion coefficients, and food availability equations may substantially influence estimates of food availability and nutrient supply. Consequently, understanding methodological changes is essential for ensuring the appropriate interpretation of temporal trends and maintaining comparability across reporting periods and countries [6,7,33].

Despite the extensive use of Portuguese FBS data in studies investigating nutrition transitions, food system sustainability, and the alignment of national food supplies with Mediterranean dietary recommendations, no study has systematically assessed the methodological evolution of Portuguese FBS or evaluated how methodological revisions may influence the interpretation of long-term trends. This represents an important knowledge gap because methodological changes can affect both temporal comparability and international benchmarking of food availability indicators [6,7,33].

Therefore, the present study aimed to conduct a comprehensive comparative analysis of the methodological evolution of Portuguese FBS between 1963 and 2024. Specifically, the study examines changes in institutional frameworks, data sources, food classification systems, nutritional indicators, conversion procedures, food loss estimation methods, and food availability equations, providing insights into the progressive alignment of Portuguese FBS with international standards and their implications for nutrition surveillance, food availability monitoring, food system monitoring, sustainability assessment, and evidence-informed policymaking [4,7,10].

## 2. Materials and Methods

### 2.1. Study Design and Data Sources

This study employed a retrospective documentary analysis to examine the methodological evolution of Portuguese FBS published by the Portuguese National Statistics Institute. Documentary analysis is a recognised qualitative research approach that enables the systematic evaluation of historical records, statistical publications, and institutional documents to identify changes in concepts, classifications, and methodological procedures over time.

Official Portuguese FBS reports corresponding to the periods 1963–1975 [28], 1980–1992 [29], 1990–1997 [30], 2012–2016 [31], 2016–2020 [8], and 2020–2024 [32] were retrieved from the Portuguese National Statistics Institute website and systematically reviewed. These reports constitute the official national source of information on food availability in Portugal and represent one of the longest-running food monitoring systems in Europe [5].

Inclusion criteria comprised all official Portuguese FBS editions published by the Portuguese National Statistics Institute between 1963 and 2024. Eligible documents consisted of official national FBS reports containing methodological information on food availability estimation, nutrient supply calculations, food classification systems, or related methodological procedures. No exclusion criteria were applied because the aim of this study was to examine the complete methodological evolution of Portuguese FBS over the entire period for which official reports were available. Consequently, all officially published FBS editions issued during the study period were included to ensure a comprehensive historical assessment. The reporting periods analysed correspond to all official Portuguese FBS editions published by the Portuguese National Statistics Institute. No official FBS reports were issued for the intervening periods; therefore, no additional documents were available for inclusion.

All available FBS editions were independently examined by two investigators, and methodological information was extracted using a standardized analytical template developed specifically for this study. The template included predefined fields covering institutional frameworks, data sources, food classification systems, nutritional indicators, food availability equations, food loss estimation procedures, conversion coefficients, nutrient calculation methods, and reporting structures. Following the independent extraction, the reviewers compared the extracted information and resolved any discrepancies through discussion until consensus was reached. The finalized dataset was subsequently organized in Microsoft Excel^®^ to facilitate cross-period comparisons and ensure consistency in data recording.

The extraction process focused on methodological characteristics that could influence food availability estimation, nutrient supply calculations, and the interpretation of long-term trends. Particular attention was given to methodological revisions associated with the implementation of the SUA framework and changes in food grouping systems when applicable.

Methodological information was systematically reviewed across all reporting periods and synthesised using a qualitative comparative approach. Differences and similarities between successive editions were identified, classified according to the predefined analytical domains described above, and interpreted based on their potential implications for food availability estimation, nutrient supply calculations, and the interpretation of long-term trends.

### 2.2. Analytical Framework

A structured analytical framework was developed to ensure a systematic and consistent comparison of Portuguese FBS across reporting periods. The framework was based on the conceptual structure of FBS and methodological recommendations established by the FAO for food supply assessment and reporting [4,5].

Three analytical domains were defined: (i) Data Sources, (ii) FBS Structure, and (iii) FBS Methodology.

The first domain, Data Sources, aimed to characterise the origin, scope, and nature of the information used to generate food availability estimates. This domain included three subcategories: (1) institutions involved in data collection, compilation, and validation; (2) types of data sources used, including agricultural production, trade statistics, stock variation, seed utilisation, animal feed, industrial use, industrial processing, food losses, and food supply data; and (3) indicators reported, classified as quantitative indicators, nutritional indicators, and socioeconomic indicators.

The second domain, FBS Structure, examined how food and nutritional information was organised and presented across reporting periods. Three subcategories were considered: (1) food classification systems and food groups included in the FBS; (2) units of measurement used to express food availability and nutrient supply; and (3) nutritional outputs and energy-related indicators reported.

The third domain, FBS Methodology, evaluated the procedures used to estimate food availability and nutritional supply. This domain comprised four subcategories: (1) food availability equations and accounting frameworks; (2) methods used to estimate food losses and waste; (3) conversion coefficients and adjustment factors applied to derive edible food availability; and (4) procedures used to calculate energy and nutrient supply.

The analytical framework provided the basis for the systematic extraction, organisation, and comparison of methodological information across all FBS editions. This approach facilitated the identification of methodological continuities, revisions, and innovations introduced over time, as well as their potential implications for the interpretation of long-term food availability trends.

### 2.3. Comparative Analysis

A qualitative comparative analysis was conducted across all reporting periods to identify and characterise methodological developments in Portuguese FBS. Comparisons were performed within and across the three analytical domains previously defined: Data Sources, FBS Structure, and FBS Methodology.

Methodological changes were identified through the systematic examination of differences in institutional frameworks, data collection systems, food classification structures, reported indicators, nutrient reporting procedures, food availability calculations, conversion coefficients, and estimation methods. Particular attention was given to methodological developments associated with successive revisions of FAO guidelines and the progressive adoption of the SUA framework [4].

To improve analytical consistency, extracted information was independently reviewed and cross-checked across reporting periods prior to synthesis. Identified methodological changes were subsequently categorised according to their potential influence on food availability estimates, nutrient supply calculations, and the comparability of long-term trends.

The findings were synthesised descriptively and presented through comparative tables and figures to facilitate the identification of methodological continuities, revisions, and key transitions in the Portuguese FBS system between 1963 and 2024. This approach enabled a comprehensive assessment of the evolution of Portuguese FBS methodologies and their implications for nutrition surveillance, food system monitoring, and the interpretation of historical food availability data.

## 3. Results

### 3.1. Evolution of Institutional Frameworks, Data Sources, and Reported Indicators

Substantial changes were identified in the institutional and methodological framework underlying Portuguese FBS between 1963 and 2024 (Table 1). Throughout all reporting periods, the Portuguese National Statistics Institute and the FAO remained central contributors to the development and methodological support of the Portuguese FBS system. However, the number and diversity of participating institutions decreased over time, reflecting a progressive transition from a broad administrative reporting structure towards a more streamlined and standardized statistical framework.

The earliest FBS editions relied on an extensive network of governmental organizations associated with agriculture, livestock production, economic coordination, and food supply management. During the 1980–1992 period, the National Institute of Health Dr. Ricardo Jorge (INSA) became involved in the compilation process, introducing a stronger public health and nutritional perspective. Subsequently, the 1990–1997 edition incorporated contributions from academic institutions and food-sector associations, representing the period with the greatest institutional diversity. From 2012 onwards, the institutional framework became more consolidated, with the Portuguese National Statistics Institute, INSA, and FAO representing the principal organizations involved in data compilation, methodological development, and validation.

Changes were also observed in the scope and structure of the information used to generate food availability estimates. Earlier FBS editions included detailed information on agricultural production, trade flows, stock variation, food distribution, animal feed, seed utilization, industrial use, food losses, and both gross and edible food supply. In contrast, more recent publications adopted a more concise and standardized reporting structure centred on food availability and nutritional indicators (Table 1). This shift reflects the increasing harmonization of Portuguese FBS with international statistical reporting practices and the growing emphasis on producing comparable indicators for nutrition surveillance and food system monitoring.

The indicators reported also evolved over time. The earliest FBS editions included quantitative, nutritional, and socioeconomic indicators, whereas later editions focused primarily on quantitative and nutritional measures. Although this change reduced the breadth of reported information, it contributed to greater standardization and facilitated comparisons across reporting periods and with international FBS datasets.

These institutional and reporting developments indicate a gradual modernization of the Portuguese FBS system. The progressive simplification of institutional arrangements, together with the standardization of data sources and reported indicators, strengthened methodological consistency and improved the capacity of Portuguese FBS to support long-term monitoring of food availability and nutritional supply.

### 3.2. Evolution of Food Classification Systems

The classification of food products evolved substantially across the study period, reflecting a progressive transition towards more detailed, standardized, and nutritionally informative food grouping systems (Tables S1–S4). The most pronounced changes occurred between the 1963–1975 and 1980–1992 editions, when broad food categories were progressively replaced by more disaggregated classifications.

For plant-based foods, the level of detail increased considerably after 1980, particularly for cereals, fruits, vegetables, pulses, and sugar products (Table S1). Earlier FBS generally reported aggregated food groups, whereas later editions distinguished a wider range of individual products and subcategories. This increased disaggregation improved the capacity of the Portuguese FBS system to capture changes in food availability patterns and the diversification of the national food supply over time.

Animal-source food classifications also underwent important modifications (Table S2). Successive editions introduced a more refined organization of meat products and expanded the classification of dairy products and fishery resources. These changes improved the nutritional relevance of the reported data and facilitated the assessment of shifts in the availability of key food groups associated with nutrition transitions.

Additional developments were observed in oils, fats, and miscellaneous food products (Table S3). Whereas the earliest FBS editions reported individual fat and oil products separately, later editions adopted broader and more standardized classifications distinguishing major categories of solid and liquid fats. These modifications contributed to greater consistency in reporting practices and improved comparability across reporting periods.

A notable structural innovation occurred in the 1990–1997 edition with the introduction of beverages as an independent food group (Table S4). Separate categories were established for fermented alcoholic beverages, other alcoholic beverages, and non-alcoholic beverages, a classification system that remained unchanged in subsequent editions. This development expanded the analytical scope of Portuguese FBS by allowing the separate monitoring of beverage availability, an increasingly important component of the national food supply.

The progressive refinement of food classification systems substantially enhanced the analytical capacity of Portuguese FBS. Greater food disaggregation improved the characterization of the national food supply, strengthened comparability with international FBS systems, and increased the usefulness of FBS data for nutrition surveillance and food system monitoring.

### 3.3. Evolution of Nutritional Supply Indicators

The nutritional information reported in Portuguese FBS expanded substantially between 1963 and 2024 (Table 2). The earliest FBS editions (1963–1975) reported only three nutritional indicators: energy, protein, and fat availability. These indicators provided a basic assessment of the nutritional adequacy of the national food supply and reflected the primary nutritional concerns of the period, which were largely focused on energy and macronutrient sufficiency.

A major development occurred in the 1980–1992 edition with the incorporation of carbohydrate availability as a routinely reported indicator. This modification expanded the nutritional scope of Portuguese FBS by enabling a more comprehensive characterization of the macronutrient profile of the national food supply. Consequently, the number of reported macronutrient indicators increased from three to four, enhancing the capacity of FBS to support nutrition surveillance and food system monitoring.

Further methodological advances were introduced in the 2012–2016 edition through the inclusion of vitamin and mineral availability indicators. This represented a substantial expansion of the nutritional information provided by Portuguese FBS and enabled the assessment of both macronutrient and micronutrient supply. The incorporation of these indicators reflected growing scientific interest in the nutritional quality of national food supplies, nutrient adequacy, and the role of food systems in supporting population health.

The micronutrient indicators incorporated from the 2012–2016 edition included vitamins A, D, and E, thiamine, riboflavin, niacin, vitamin B6, folate, vitamin B12, and vitamin C, together with the minerals sodium, potassium, calcium, phosphorus, magnesium, iron, zinc, copper, manganese, selenium, and iodine. These additions substantially expanded the nutritional information available through Portuguese FBS.

As a result of these developments, Portuguese FBS progressively expanded the range of reported nutritional indicators, increasing from three categories in 1963–1975 to six categories in 2020–2024 (Table 2). This progression illustrates the gradual transition of Portuguese FBS from instruments primarily designed to monitor food supply towards increasingly comprehensive tools for nutritional assessment and public health monitoring.

The expansion of nutritional reporting increased the analytical value of Portuguese FBS for research and policymaking. By incorporating a broader range of nutrients, more recent FBS editions provide a more comprehensive characterization of the nutritional capacity of the Portuguese food system and facilitate the evaluation of long-term changes in food availability, the nutritional composition of the national food supply, and nutrient availability. These developments further strengthened the role of Portuguese FBS as instruments for nutrition surveillance and food system monitoring, supporting evidence-informed food and nutrition policies while improving alignment with contemporary international approaches to food system monitoring.

### 3.4. Evolution of Food Availability Equations and Food Loss Estimation Methods

Important methodological developments were identified in the equations used to estimate food availability across Portuguese FBS (Table 3). The most substantial changes occurred after 2012, reflecting the progressive adoption of more comprehensive accounting procedures and closer alignment with FAO methodological recommendations.

The 1980–1992 and 1990–1997 editions employed a relatively simple food balance equation based on four principal components: production, imports, exports, and stock variation. This approach provided a general estimate of food availability but did not explicitly account for several non-food uses that may substantially influence the quantity of food ultimately available for human consumption.

From the 2012–2016 edition onwards, food availability calculations became considerably more comprehensive. The revised framework incorporated additional components including seed use, eggs for incubation, animal feed, industrial utilization, industrial processing, food losses, and stock variation. As a result, the number of components explicitly included in food availability calculations increased from four to nine, representing a substantial methodological expansion of the accounting framework. However, the methodological documentation accompanying post-2012 FBS editions provides less detailed information regarding food loss estimation than earlier editions. It was therefore not possible to determine whether the underlying estimation procedures changed or whether only the level of methodological reporting differed. This limitation should be taken into account when interpreting long-term trends in food availability across reporting periods.

The incorporation of these additional elements improved the capacity of Portuguese FBS to distinguish food intended for human consumption from quantities diverted to alternative uses. This development reduced the risk of overestimating food availability and enhanced the consistency of food supply estimates with contemporary international standards. The revised approach also strengthened the comparability of Portuguese FBS with FBS systems implemented in other countries following FAO recommendations.

Methodological differences were also observed in the treatment of food losses and waste. The earliest FBS editions explicitly reported the use of product-specific loss coefficients that varied according to food category (Table 3). These coefficients were applied to estimate reductions occurring during storage, distribution, and processing stages. In contrast, more recent editions incorporated food losses directly within the food availability equation but provided less detailed information regarding the procedures used to estimate such losses.

The evolution of food loss accounting reflects broader methodological changes in FBS compilation. Earlier approaches focused on documenting adjustment procedures, whereas more recent frameworks emphasize integrated accounting systems in which losses are considered as one component of overall food utilization. Although differences in reporting detail limit direct comparisons of loss estimation procedures across periods, the inclusion of food losses within contemporary accounting frameworks represents an important methodological advancement.

These methodological changes also have important implications for the interpretation of historical trends. Earlier FBS editions provided more detailed documentation of the application of product-specific loss coefficients, whereas more recent editions incorporate food losses within a broader accounting framework but provide less detailed methodological documentation. As a result, differences observed across reporting periods may partly reflect changes in food loss estimation procedures rather than actual changes in food availability. This aspect should be considered when interpreting long-term trends based on Portuguese FBS.

These findings indicate that the transition from simplified food balance equations to more comprehensive accounting frameworks constitutes one of the most significant methodological developments identified in Portuguese FBS. The progressive incorporation of additional utilization categories and food loss adjustments enhanced the accuracy, methodological standardization, and international comparability of food availability estimates while strengthening the usefulness of FBS for nutrition surveillance and food system monitoring.

### 3.5. Evolution of Conversion Coefficients and Nutrient Calculation Methods

Methodological refinements were also identified in the procedures used to estimate edible food availability and nutrient supply (Table 4). These developments primarily involved changes in the application of conversion coefficients and adjustment factors designed to transform gross food availability into estimates more closely reflecting food available for human consumption.

The earliest FBS edition (1963–1975) relied directly on values derived from the Portuguese Food Composition Table to estimate edible food availability and nutrient supply. Subsequent editions introduced increasingly explicit procedures for adjusting gross food supply data through the application of food-specific conversion coefficients. During the 1980–1992 period, percentage coefficients derived from the Portuguese Food Composition Table were applied to gross per capita food supply estimates to calculate edible availability.

Further methodological refinement was observed from the 2012–2016 edition onwards, when product-specific edible portion coefficients were systematically applied to gross food availability estimates. These coefficients allowed more detailed adjustments according to the characteristics of individual food products and contributed to improved estimates of edible food supply. Similar procedures were maintained in the 2016–2020 and 2020–2024 editions, indicating increasing methodological standardization in the estimation of edible food availability.

The adoption of food-specific edible portion coefficients enhanced the capacity of Portuguese FBS to account for differences between gross and edible food quantities. By incorporating adjustments for inedible fractions, these procedures improved the accuracy of food availability estimates and reduced potential biases associated with the use of unadjusted food supply data.

In contrast to the changes observed in conversion procedures, energy calculation methods remained remarkably stable throughout the study period. All FBS editions used the Portuguese Food Composition Table as the primary reference source for estimating energy and nutrient availability (Table 4). This continuity contributed to maintaining consistency in nutrient supply calculations despite the methodological developments observed in other components of the FBS system.

The coexistence of methodological refinement in edible portion estimation and stability in nutrient calculation procedures highlights a balanced evolution of the Portuguese FBS system. While increasingly sophisticated conversion methods improved the precision of food availability estimates, the continued use of a common nutritional reference source supported the comparability of energy and nutrient supply indicators across reporting periods.

### 3.6. Summary of Major Methodological Transitions

**Figure 1:**
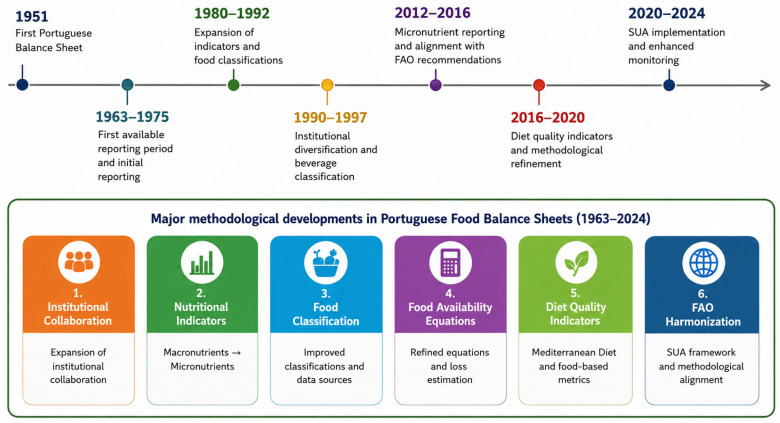
Six decades of methodological evolution in Portuguese Food Balance Sheets: key milestones and major developments (1963–2024). Created by the authors based on the comparative analysis conducted in this study.

A synthesis of the principal methodological developments identified across all reporting periods is presented in Figure 1. Portuguese FBS evolved progressively from relatively simple food supply accounting systems towards more comprehensive and standardized frameworks for food availability assessment and nutrition surveillance. Major methodological developments included the refinement of food classification systems, the expansion of nutritional indicators, the adoption of more comprehensive food availability equations, the progressive standardization of conversion procedures, and increasing alignment with FAO methodological recommendations.

A major milestone in this evolution was the adoption of accounting procedures consistent with the SUA framework, which incorporated a broader range of food utilization categories within food availability calculations. Figure 1 also highlights other important methodological developments, including the expansion of nutritional reporting, the introduction of dedicated beverage categories, and successive refinements in food availability estimation procedures across reporting periods.

These findings illustrate the progressive methodological evolution of Portuguese FBS between 1963 and 2024 and provide a framework for interpreting methodological differences across reporting periods.

## 4. Discussion

### 4.1. Principal Findings

This study provides the first comprehensive assessment of the methodological evolution of Portuguese FBS over more than six decades. The findings indicate a progressive methodological evolution of Portuguese FBS, reflecting advances in food supply monitoring and the increasing alignment of national methodologies with international standards established by the FAO [4,5,7]. Major developments were identified in institutional frameworks, food classification systems, nutritional indicators, food availability equations, and conversion procedures, illustrating the continuous refinement of the Portuguese FBS system.

One of the most important developments was the progressive harmonization of Portuguese FBS with FAO recommendations through increasingly standardized accounting procedures and the adoption of approaches consistent with the Supply Utilization Accounts (SUA) framework. These methodological revisions may have improved methodological consistency and international comparability, although their quantitative impact on food availability estimates was beyond the scope of the present study [4,5].

A progressive expansion of the nutritional information reported by Portuguese FBS was also observed, from three nutritional indicators (energy, protein, and fat) in the earliest editions to a broader set including carbohydrates, vitamins, and minerals in more recent editions. This evolution reflects broader developments in nutritional science and the increasing emphasis on more comprehensive assessments of nutrient availability within national food supplies [9,10,11].

Another important methodological development was the increasing complexity of food availability estimation procedures. Successive editions progressively incorporated additional accounting components, including animal feed, seed utilization, industrial uses, industrial processing, and food losses, reflecting the adoption of more comprehensive accounting frameworks. Although these methodological revisions were intended to improve the completeness of food availability estimates, they also introduced methodological discontinuities that should be considered when comparing data across reporting periods [4,5,7].

The findings demonstrate that Portuguese FBS have undergone substantial methodological evolution over time. This evolution may strengthen the usefulness of FBS for nutrition surveillance, food system monitoring, and evidence-informed policymaking, provided that major methodological transitions are carefully considered when interpreting long-term food availability trends.

### 4.2. Implications for the Interpretation of Long-Term Food Availability Trends

One of the most important implications of the findings concerns the interpretation of long-term trends derived from Portuguese FBS. Although FBS are frequently used to evaluate long-term changes in national food availability and to support research on nutrition transitions and food system performance, the findings suggest that methodological revisions may influence food availability estimates independently of actual changes in the underlying food system [6,7].

The evolution of food availability equations provides a clear example of this challenge. Earlier Portuguese FBS were based on relatively simple accounting procedures that considered a limited number of food flow components. More recent editions adopted expanded accounting frameworks that explicitly incorporate animal feed, seed utilization, industrial uses, industrial processing, and food losses. Although these revisions were intended to improve the accuracy of food availability estimates, they also introduce potential discontinuities when comparing data across reporting periods [4,5]. Consequently, apparent increases or decreases in food availability may partly reflect methodological modifications rather than genuine changes in food supply patterns.

The incorporation of food losses into food availability calculations represents another important methodological development. Food losses can account for a substantial proportion of the food supply chain and may vary considerably across food groups and production systems. Earlier Portuguese FBS relied on food-specific loss coefficients, whereas more recent editions integrated food losses directly into broader accounting procedures. Although these approaches aim to provide more realistic estimates of food available for human consumption, differences in loss estimation methods may affect the comparability of historical series [4,5,7].

Changes in food classification systems and nutritional reporting procedures may also influence the interpretation of long-term trends. The progressive disaggregation of food groups and the expansion of nutritional indicators may improve the analytical capacity of FBS but can complicate direct comparisons between reporting periods. Similar challenges have been identified in other national and international food supply datasets, where revisions in classification systems, nutrient databases, and estimation procedures have generated differences in reported food availability and nutrient supply over time [6,33].

These findings highlight the importance of considering methodological context when interpreting historical FBS data. Long-term analyses based on FBS should account for major methodological transitions, particularly when evaluating trends spanning multiple decades or comparing estimates generated under different accounting frameworks. Failure to consider these changes may lead to inaccurate conclusions regarding the magnitude or direction of nutrition transitions and food system developments.

Rather than being interpreted solely as a limitation, methodological evolution reflects the continuous effort to improve the accuracy, relevance, and policy usefulness of food supply statistics. Nevertheless, awareness of these methodological transitions is essential for researchers, policymakers, and public health professionals seeking to use FBS data for longitudinal analyses, international comparisons, or evidence-based decision-making.

From a practical perspective, researchers and policymakers should consider major methodological transitions when interpreting long-term trends derived from Portuguese FBS. Comparisons across reporting periods characterized by substantial methodological revisions, particularly those introduced after 2012, should be interpreted with caution. Where major methodological discontinuities exist, FBS data should be interpreted alongside complementary sources of information, such as national dietary surveys, household budget surveys, or food purchase datasets.

It is also important to distinguish between methodological improvement and methodological discontinuity. Several methodological revisions introduced in successive Portuguese FBS editions, particularly the expansion of the food availability equation after 2012, were intended to improve the completeness and accuracy of food availability estimates. However, these revisions also introduced methodological discontinuities that may limit direct comparisons with earlier editions. Therefore, methodological improvements should not necessarily be interpreted as evidence of uninterrupted comparability across the entire time series. Increases or decreases in food availability and nutrient supply observed across the 2012 methodological transition should not automatically be interpreted as reflecting true changes in the national food supply, as part of the observed variation may result from methodological revisions rather than actual changes in food availability.

### 4.3. International Harmonization and the SUA Framework

The findings of this study indicate that one of the most significant developments in the evolution of Portuguese FBS has been their progressive alignment with international methodological standards established by the FAO. This process reflects broader efforts to strengthen methodological consistency and international comparability of food supply statistics across countries, potentially enhancing the value of FBS for global food system monitoring and policy evaluation [4,5].

Historically, national FBS were often compiled using country-specific procedures, data sources, and accounting conventions. Although these approaches provided valuable information for national monitoring purposes, methodological differences frequently limited the comparability of food availability estimates across countries and over time. To address these challenges, the FAO progressively developed standardized methodological frameworks aimed at harmonizing food balance compilation procedures and improving the coherence of international food supply datasets [4,5].

A particularly important milestone in this process was the adoption of the SUA framework. Unlike traditional food balance approaches, SUA is intended to provide a more comprehensive representation of food flows by explicitly accounting for production, imports, exports, stock variation, animal feed, seed utilization, industrial uses, processing activities, and food losses within a unified accounting structure [4]. The incorporation of these components is intended to improve the completeness and consistency of food availability calculations and reduce the likelihood of inconsistencies arising from incomplete accounting procedures.

The transition towards SUA-based methodologies observed in the most recent Portuguese FBS is consistent with international trends in food supply monitoring. Previous studies have highlighted the importance of methodological harmonization for improving the reliability and interpretability of national food availability estimates, particularly when such data are used for cross-country comparisons, food system monitoring, and global nutrition surveillance initiatives [6,7,10]. Greater methodological consistency may also facilitate the integration of FBS data into broader analytical frameworks examining food security, the nutritional characteristics of national food supplies, sustainability, and food system resilience.

The adoption of internationally harmonized procedures does not eliminate all limitations associated with FBS. As indirect measures of food availability, FBS remain unable to capture individual-level dietary intake, intra-household food distribution, or socioeconomic inequalities in food access [7]. Nevertheless, methodological harmonization may improve the comparability of food supply statistics and strengthen their value as population-level indicators of food system performance.

The progressive alignment of Portuguese FBS with FAO recommendations and SUA principles therefore represents more than a technical adjustment in statistical methodology. It reflects the integration of Portuguese food monitoring systems into an increasingly standardized international framework and may strengthen the contribution of FBS to national policymaking, nutrition surveillance, food security assessment, and sustainable food system monitoring.

### 4.4. Expanding Applications of Food Balance Sheets

The methodological developments identified in Portuguese FBS have broadened their potential applications beyond their traditional role as instruments for monitoring national food supplies. While FBS were originally developed to assess food availability and support food security planning, they are increasingly used to investigate nutrition transitions, characterize the nutritional composition of national food supplies, assess food system sustainability, and examine the relationship between food availability and population health outcomes [7,10]. Previous studies have documented substantial shifts in food availability across many countries, including increased availability of animal-source foods, vegetable oils, added sugars, and ultra-processed products, changes that have been associated with rising levels of obesity and other chronic diseases [11,12,13,14]. In Portugal, similar trends have been reported over recent decades, reflecting broader transformations in food environments and consumption patterns [21,25,26]. The methodological developments identified in the present study may strengthen the capacity of Portuguese FBS to support the monitoring and interpretation of such transitions.

The methodological developments identified in Portuguese FBS also expand their application to food system sustainability assessments. The incorporation of additional nutritional indicators and the progressive alignment with international standards may strengthen the value of Portuguese FBS for evaluating the relationship between food availability, the nutritional quality of the national food supply, and sustainable food systems, as well as for assessing the nutritional adequacy of national food supplies [15,16,17]. These methodological advances may further enhance the value of Portuguese FBS for evidence-informed nutrition and food system policies.

Within Mediterranean countries, FBS have played an important role in documenting long-term changes in food availability. Several studies have reported increasing availability of foods associated with more Westernized food supply patterns, indicating a progressive departure of national food supplies from traditional Mediterranean dietary characteristics [20,21,22]. These findings illustrate the value of FBS for supporting research on nutrition transitions and the alignment of national food supplies with Mediterranean dietary recommendations [20,21,22,23,24]. In the Portuguese context, the incorporation of additional nutritional indicators has further expanded the analytical scope of FBS by supporting a more comprehensive characterization of the nutritional composition of the national food supply. Together with the progressive alignment of Portuguese FBS with international methodological standards, these developments may strengthen the use of FBS as sources of information for nutrition surveillance, food system monitoring, and evidence-informed decision-making across nutrition, agriculture, public health, and sustainability.

### 4.5. Strengths and Limitations

This study has several strengths. To our knowledge, it is the first comprehensive assessment of the methodological evolution of Portuguese FBS over more than six decades. By systematically examining all available Portuguese FBS editions published between 1963 and 2024, the study provides a unique historical perspective on the development of food availability monitoring in Portugal. The structured analytical framework employed in this study enabled the consistent comparison of institutional arrangements, data sources, food classification systems, nutritional indicators, food availability equations, and conversion methodologies across reporting periods. Furthermore, the study contributes to the international literature on FBS by highlighting how methodological revisions may influence the interpretation of long-term food availability trends and by documenting the progressive alignment of a national FBS system with FAO recommendations and the SUA framework [4,5].

Several limitations should also be acknowledged. First, the study relied exclusively on methodological information reported in official FBS publications. Consequently, undocumented methodological decisions, data processing procedures, or revisions implemented internally by statistical agencies could not be evaluated. Second, the analysis focused on the description and comparison of methodological approaches and did not quantify the magnitude of the effects that individual methodological changes may have had on food availability or nutrient supply estimates. Therefore, the implications discussed throughout this study should be interpreted as potential consequences of the identified methodological developments rather than empirically demonstrated effects. Future studies could address this limitation by applying alternative methodologies to historical datasets and assessing the extent to which methodological revisions influence reported trends over time. In addition, although Portuguese FBS consistently report nutrient availability estimates, the specific editions of the Portuguese Food Composition Table used for nutrient calculations were not systematically documented across all FBS reports. As a result, the potential influence of revisions to the Portuguese Food Composition Table on long-term nutrient supply estimates could not be fully assessed and should be considered when interpreting temporal trends.

Additional limitations are inherent to FBS themselves. As indirect measures of food availability, FBS estimate the quantity of food theoretically available for human consumption rather than actual dietary intake. They do not capture individual food consumption patterns, intra-household food distribution, food purchases, food waste occurring at household level, or differences in food access among population subgroups [6,7]. Consequently, FBS should be interpreted as population-level indicators of food availability and food system performance rather than direct measures of dietary behaviour or nutritional status.

Despite these limitations, FBS remain one of the most valuable and widely available sources of information for monitoring national food supplies and evaluating long-term changes in food availability [7]. The methodological developments identified in this study suggest that contemporary Portuguese FBS may provide a more comprehensive basis for food system assessment and policy development than earlier editions. Understanding these methodological transitions is important for the appropriate interpretation of historical food availability data and for the informed use of FBS in future research and decision-making processes.

## 5. Conclusions

Portuguese FBS have undergone substantial methodological evolution between 1963 and 2024, reflecting advances in nutritional science, improvements in statistical systems, and the progressive alignment of national food monitoring practices with FAO recommendations. Major developments included the expansion of food classification systems, the incorporation of additional nutritional indicators, the refinement of food availability equations, the adoption of product-specific conversion procedures, and the implementation of methodologies consistent with the SUA framework. These changes progressively expanded the scope of Portuguese FBS and contributed to the methodological evolution of national food availability monitoring.

The findings indicate that methodological revisions should be considered when interpreting and comparing long-term food availability data, as they may influence the comparability of historical series. Consequently, historical analyses based on FBS should consider major methodological transitions when evaluating long-term changes in food availability, nutrition transitions, and food system developments across reporting periods.

Although FBS remain indirect measures of food availability and cannot replace methods for assessing individual food consumption, they continue to represent valuable tools for population-level food system assessment, food security evaluation, and evidence-informed policymaking. The progressive harmonization of Portuguese FBS with international standards may enhance their relevance for both national and international research and strengthen their contribution to food system monitoring. The present study systematically identified and described the major methodological developments in Portuguese FBS but did not quantify the extent to which individual methodological changes influenced food availability or nutrient supply estimates. By documenting six decades of methodological evolution, this study provides a reference framework for the interpretation of Portuguese FBS and for future methodological research.

### Future Research

Future research should prioritize comprehensive documentation of methodological revisions, continued methodological harmonization, and the standardization of food availability indicators. Such advances may further improve the comparability, reliability, and policy relevance of FBS and support their use in nutrition, public health, and sustainable food system research.

Future studies should also quantify the impact of individual methodological revisions on food availability and nutrient supply estimates and explore approaches for harmonizing historical FBS series to improve the comparability of long-term trends. Particular attention should be given to evafbsluating the effects of changes in food availability equations, food loss estimation procedures, food classification systems, nutrient reporting, and food composition data on historical food availability and nutrient supply estimates.

In addition, the adoption of a stable and internationally harmonized food classification system in future FBS compilations may further improve the interoperability of Portuguese FBS with international datasets and facilitate robust comparative analyses across countries and reporting periods.

In the absence of regularly updated national dietary surveys, Portuguese FBS may continue to represent an important national source of information for monitoring long-term changes in food availability and supporting food system analyses. Future methodological developments should therefore seek to improve the consistency, documentation, and comparability of Portuguese FBS while preserving the continuity and interpretability of historical time series.

## Figures and Tables

**Table 1:** Evolution of institutional frameworks, data sources, reported indicators, and major methodological developments in Portuguese Food Balance Sheets (1963–2024).

Period	Main Institutions Involved	Data Sources Included	Indicators Reported	Major Methodological Development
1963–1975 [28]	INE, agricultural and livestock governmental agencies, economic coordination bodies, National FAO Commission	Production, trade, stock variation, food distribution, animal feed, seed use, industrial use, losses, gross and edible food supply	Quantitative, nutritional, and socioeconomic indicators	Establishment of the Portuguese FBS system and broad institutional participation
1980–1992 [29]	INE, INSA, FAO	Production, trade, stock variation, food distribution, animal feed, seed use, industrial use, losses, gross and edible food supply	Quantitative and nutritional indicators	Integration of public health expertise through INSA participation
1990–1997 [30]	INE, INSA, FAO, academic institutions, food-sector associations	Production, trade, stock variation, food distribution, animal feed, seed use, industrial use, losses, gross and edible food supply	Quantitative and nutritional indicators	Expansion of institutional collaboration and technical support
2012–2016 [31]	INE, INSA, FAO	Food availability and trade-based data integrated within a revised FBS framework	Quantitative and nutritional indicators	Introduction of a revised methodological framework and greater standardization
2016–2020 [8]	INE, INSA, FAO	Food availability and trade-based data integrated within a revised FBS framework	Quantitative and nutritional indicators	Consolidation of standardized reporting procedures
2020–2024 [32]	INE, INSA, FAO	Food availability and trade-based data integrated within a revised FBS framework	Quantitative and nutritional indicators	Alignment with contemporary FAO recommendations and implementation of SUA-based procedures

Abbreviations: FAO, Food and Agriculture Organization of the United Nations; FBS, Food Balance Sheets; INE, Portuguese National Statistics Institute; INSA, National Institute of Health Dr. Ricardo Jorge; SUA, Supply and Utilization Accounts.

**Table 2:** Evolution of nutritional supply indicators reported in Portuguese Food Balance Sheets (1963–2024).

Nutritional Indicator	1963–1975 [28]	1980–1992 [29]	1990–1997 [30]	2012–2016 [31]	2016–2020 [8]	2020–2024 [32]
Energy	✓	✓	✓	✓	✓	✓
Protein	✓	✓	✓	✓	✓	✓
Fat	✓	✓	✓	✓	✓	✓
Carbohydrates	–	✓	✓	✓	✓	✓
Vitamins	–	–	–	✓	✓	✓
Minerals	–	–	–	✓	✓	✓
Total nutritional indicators reported	3	4	4	6	6	6

Note: “✓” indicates that the nutritional indicator was reported in the corresponding edition of the Portuguese Food Balance Sheets; “–” indicates that the indicator was not reported.

**Table 3:** Evolution of food availability equations and food loss estimation methods in Portuguese Food Balance Sheets (1963–2024).

FBS Period	Food Availability Equation	Number of Components Included	Food Loss and Waste Estimation	Major Methodological Development
1963–1975 [28]	Not explicitly reported	N/A	Estimated using product-specific coefficients varying according to food category	First documented use of food-specific loss coefficients
1980–1992 [29]	Production + Imports − Exports − Stock Variation	4	Estimated using product-specific coefficients varying according to food category	Introduction of an explicit food balance equation
1990–1997 [30]	Production + Imports − Exports − Stock Variation	4	Estimated using product-specific coefficients varying according to food category	Maintenance of simplified food balance accounting framework
2012–2016 [31]	Production + Imports − Exports − Seeds/Eggs for Incubation − Animal Feed − Industrial Use − Industrial Processing − Losses − Stock Variation	9	Not explicitly reported	Adoption of a substantially expanded accounting framework aligned with FAO recommendations
2016–2020 [8]	Production + Imports − Exports − Seeds/Eggs for Incubation − Animal Feed − Industrial Use − Industrial Processing − Losses − Stock Variation	9	Not explicitly reported	Consolidation of expanded food availability accounting procedures
2020–2024 [32]	Production + Imports − Exports − Seeds/Eggs for Incubation − Animal Feed − Industrial Use − Industrial Processing − Losses − Stock Variation	9	Not explicitly reported	Alignment with SUA principles and contemporary FAO methodology

Abbreviations: FAO, Food and Agriculture Organization of the United Nations; FBS, Food Balance Sheets; SUA, Supply and Utilization Accounts.

**Table 4:** Evolution of conversion coefficients and nutrient calculation methods in Portuguese Food Balance Sheets (1963–2024).

FBS Period	Conversion Coefficients	Energy Calculation Method	Level of Methodological Refinement
1963–1975 [28]	Values derived directly from the Portuguese Food Composition Table	Portuguese Food Composition Table	Basic estimation approach
1980–1992 [29]	Percentage coefficients from the Portuguese Food Composition Table applied to gross per capita food supply to estimate edible availability	Portuguese Food Composition Table	Introduction of standardized conversion coefficients
1990–1997 [30]	Not explicitly reported	Portuguese Food Composition Table	Limited methodological information available
2012–2016 [31]	Product-specific edible portion coefficients applied to gross per capita food supply, based on the Portuguese Food Composition Table	Portuguese Food Composition Table	Enhanced estimation of edible food availability
2016–2020 [8]	Product-specific edible portion coefficients applied to gross per capita food supply, based on the Portuguese Food Composition Table	Portuguese Food Composition Table	Consolidation of standardized edible portion adjustments
2020–2024 [32]	Product-specific edible portion coefficients applied to gross per capita food supply, based on the Portuguese Food Composition Table	Portuguese Food Composition Table	Continued application of refined conversion procedures

Abbreviation: FBS, Food Balance Sheets.

## Data Availability

The original contributions presented in this study are included in the article/Supplementary Materials. Further inquiries can be directed to the corresponding author.

## Supplementary Materials

The following supporting information can be downloaded at: https://www.mdpi.com/article/10.3390/nu18142341/s1. Table S1: Changes in plant-based food classification systems across Portuguese Food Balance Sheets from 1963 to 2024; Table S2: Changes in animal-source food classification systems across Portuguese Food Balance Sheets from 1963 to 2024; Table S3: Changes in the classification of fats, oils, oilseeds, and miscellaneous food products across Portuguese Food Balance Sheets from 1963 to 2024; Table S4. Changes in beverage categories and reporting structures across Portuguese Food Balance Sheets from 1963 to 2024.
