# Supplementary material for: Six Decades of Methodological Evolution in Portuguese Food Balance Sheets: Implications for Nutrition Surveillance and Food System Monitoring
Source: Nutrients. 2026 Jul 16;18(14):2341. doi: 10.3390/nu18142341 (PMC13414670; doi:10.3390/nu18142341)
Supplement: Supplementary file 1 [file nutrients-18-02341-s001.zip › nutrients-4396687-supplementary.pdf]

Supplementary Materials

Table S1. Changes in plant-based food classification systems across Portuguese Food Balance Sheets from 1963 to 2024.

Food Category	1963–1975	1980–1992	1990–1997	2012–2016	2016–2020	2020–2024
**Cereals and Rice**	Wheat; Rice in husk; Other cereals (corn, barley, others including oat flakes)	Wheat; Rice in husk; Wheat bran and other products; Corn; Rye; Barley; Oats and other cereals	Wheat; Rice in husk; Wheat bran and other products; Corn; Rye; Oats and other cereals	Wheat; Rice in husk; Wheat bran and other products; Corn; Rye; Oats and other cereals	Wheat; Rice in husk; Wheat bran and other products; Corn; Rye; Oats and other cereals	Wheat; Rice in husk; Wheat bran and other products; Corn; Rye; Oats and other cereals
**Roots and Tubers**	Potato; Cassava and tapioca; Starch and flours	Potato; Other roots and tubers	Potato; Other roots and tubers	Potato; Other roots and tubers	Potato; Other roots and tubers	Potato; Other roots and tubers
**Sugars**	Sugar; Honey	Sucrose and other sugars; Honey	Sucrose and other sugars; Honey	Sucrose and other sugars; Honey	Sucrose and other sugars; Honey	Sucrose and other sugars; Honey
**Legumes**	Chickpeas; Dry beans; Groundnuts in shell; Others (lentils, dried peas, soybeans)	Dry beans; Chickpeas	Dry beans; Chickpeas	Dry beans; Chickpeas	Dry beans; Chickpeas	Dry beans; Chickpeas
**Horticultural Products**	Horticultural products	Tomato; Carrot; Other horticultural products	Tomato; Carrot; Other horticultural products	Tomato; Carrot; Other horticultural products	Tomato; Carrot; Other horticultural products	Tomato; Carrot; Other horticultural products
**Fruits, Including Olives**	Bananas and pineapples; Other fresh fruits; Dried fruits (including almonds, walnuts, hazelnuts in shell, chestnuts and carob); Dried fruits (including raisins, grapes, prunes and dates); Olives	Fresh fruit excluding citrus fruits (apples, pears, peaches, table grapes and other fresh fruits); Citrus fruits (oranges and other citrus fruits); Nuts	Fresh fruit excluding citrus fruits (apples, pears, peaches, table grapes and other fresh fruits); Citrus fruits (oranges and other citrus fruits); Nuts; Olives	Fresh fruit excluding citrus fruits (apples, pears, peaches, table grapes and other fresh fruits); Citrus fruits (oranges and other citrus fruits); Nuts; Olives	Fresh fruit excluding citrus fruits (apples, pears, peaches, table grapes and other fresh fruits); Citrus fruits (oranges and other citrus fruits); Nuts; Olives	Fresh fruit excluding citrus fruits (apples, pears, peaches, table grapes and other fresh fruits); Citrus fruits (oranges and other citrus fruits); Nuts; Olives

Note: The table presents the complete classification of plant-based food categories reported in Portuguese Food Balance Sheets across the study period. Changes primarily occurred between the 1963–1975 and 1980–1992 editions, reflecting a progressive standardization of food grouping systems and increasing alignment with international Food Balance Sheet methodologies.

Table S2. Changes in animal-source food classification systems across Portuguese Food Balance Sheets from 1963 to 2024.

Food Category	1963–1975	1980–1992	1990–1997	2012–2016	2016–2020	2020–2024
**Meat and Edible Offal**	Adult cattle; Young cattle; Sheep and goats; Pigs; Poultry; Game; Offal; Others (horse)	Beef; Pork; Poultry meat; Sheep and goat meat; Other meats; Edible offal	Beef; Pork; Poultry meat; Sheep and goat meat; Other meats; Edible offal	Beef; Pork; Poultry meat; Sheep and goat meat; Other meats; Edible offal	Beef; Pork; Poultry meat; Sheep and goat meat; Other meats; Edible offal	Beef; Pork; Poultry meat; Sheep and goat meat; Other meats; Edible offal
**Eggs**	Eggs	Eggs	Eggs	Eggs	Eggs	Eggs
**Milk and Dairy Products**	Milk; Cheese	Milk; Yogurts and other fermented milks; Cream; Whole and semi-skimmed milk powder; Skimmed milk powder; Cheese; Other dairy products	Milk; Yogurts and other fermented milks; Cream; Milk powder; Cheese; Other dairy products	Milk; Yogurts and other fermented milks; Cream; Milk powder; Cheese; Other dairy products	Milk; Yogurts and other fermented milks; Cream; Milk powder; Cheese; Other dairy products	Milk; Yogurts and other fermented milks; Cream; Milk powder; Cheese; Other dairy products
**Fish and Seafood Products**	Fresh fish; Dried cod; Crustaceans; Mollusks	Fresh, chilled, frozen, or canned fish; Cod and other dried, salted, smoked, or brined fish; Crustaceans and Mollusks	Fresh, chilled, frozen, or canned fish; Cod and other dried, salted, smoked, or brined fish; Crustaceans and Mollusks	Fresh, chilled, frozen, or canned fish; Cod and other dried, salted, smoked, or brined fish; Crustaceans and Mollusks	Fresh, chilled, frozen, or canned fish; Cod and other dried, salted, smoked, or brined fish; Crustaceans and Mollusks	Fresh, chilled, frozen, or canned fish; Cod and other dried, salted, smoked, or brined fish; Crustaceans and Mollusks

Note: The table presents the complete classification of animal-source food categories reported in Portuguese Food Balance Sheets between 1963 and 2024. The most substantial changes occurred between the 1963–1975 and 1980–1992 editions, particularly for dairy products and fish categories, reflecting increased disaggregation and standardization of food classifications. Subsequent editions showed a high degree of structural stability, facilitating longitudinal comparisons of food availability estimates.

Table S3. Changes in the classification of fats, oils, oilseeds, and miscellaneous food products across Portuguese Food Balance Sheets from 1963 to 2024.

Food Category	1963–1975	1980–1992	1990–1997	2012–2016	2016–2020	2020–2024
**Oilseeds and Nuts**	Not reported as a separate category	Reported as a separate category	Not reported as a separate category	Not reported as a separate category	Not reported as a separate category	Not reported as a separate category
**Oils and Fats**	Olive oil; Edible oils (including food oils and other edible oils); Butter; Margarine; Lard; Bacon fat	Solid fats (butter, margarine and similar products, lard, bacon fat and other fats); Liquid fats (olive oil and other vegetable oils)	Solid fats (butter, margarine and similar products, lard, bacon fat and other fats); Oils and liquid fats (olive oil and other refined vegetable oils)	Solid fats (butter, margarine and similar products, lard, bacon fat and other fats); Liquid fats (olive oil and other refined vegetable oils)	Solid fats (butter, margarine and similar products, lard, bacon fat and other fats); Liquid fats (olive oil and other refined vegetable oils)	Solid fats (butter, margarine and similar products, lard, bacon fat and other fats); Liquid fats (olive oil and other refined vegetable oils)
**Other Food Products**	Spices; Coconut and cocoa; Roasted products (including coffee and roasted substitutes such as chicory, barley, and rye); Teas; Non-alcoholic beverages (soft drinks, syrups, mineral waters); Alcoholic beverages (wine, beer)	Cocoa and chocolates; Coffee, coffee mixtures, and substitutes	Cocoa and chocolates; Coffee, coffee mixtures, and substitutes	Cocoa and chocolates; Coffee, coffee mixtures, and coffee substitutes	Cocoa and chocolates; Coffee, coffee mixtures, and coffee substitutes	Cocoa and chocolates; Coffee, coffee mixtures, and coffee substitutes

Note: The classification of oils and fats evolved substantially between the 1963–1975 and 1980–1992 editions, shifting from individual fat and oil products to broader categories of solid and liquid fats. The category "Oilseeds and Nuts" was explicitly reported only in the 1980–1992 Food Balance Sheet. The category "Other Food Products" underwent considerable simplification after 1975, with beverage-related products subsequently reported in dedicated beverage categories rather than within miscellaneous food products.

Table S4. Changes in beverage categories and reporting structures across Portuguese Food Balance Sheets from 1963 to 2024.

Beverage Category	1963–1975	1980–1992	1990–1997	2012–2016	2016–2020	2020–2024
**Fermented Alcoholic Beverages**	Included within the category "Other Food Products"	Not reported as a separate category	Wine and derivatives; Beer; Other fermented beverages	Wine and derivatives; Beer; Other fermented beverages	Wine and derivatives; Beer; Other fermented beverages	Wine and derivatives; Beer; Other fermented beverages
**Other Alcoholic Beverages**	Included within the category "Other Food Products"	Not reported as a separate category	Spirits (40% alcohol by volume); Liqueurs (25% alcohol by volume); Other spirits (40% alcohol by volume)	Spirits (40% alcohol by volume); Liqueurs (25% alcohol by volume); Other spirits (40% alcohol by volume)	Spirits (40% alcohol by volume); Liqueurs (25% alcohol by volume); Other spirits (40% alcohol by volume)	Spirits (40% alcohol by volume); Liqueurs (25% alcohol by volume); Other spirits (40% alcohol by volume)
**Non-Alcoholic Beverages**	Included within the category "Other Food Products"	Not reported as a separate category	Water; Soft drinks; Fruit juices, nectars and syrups	Water; Soft drinks; Fruit juices, nectars and syrups	Water; Soft drinks; Fruit juices, nectars and syrups	Water; Soft drinks; Fruit juices, nectars and syrups

Abbreviation: FBS, Food Balance Sheets.

Note: Beverage categories were not reported separately in the 1963–1975 Food Balance Sheet and were included within the broader category "Other Food Products". During the 1980–1992 period, beverages were not presented as distinct categories. A dedicated beverage classification system was introduced in the 1990–1997 edition, distinguishing fermented alcoholic beverages, other alcoholic beverages, and non-alcoholic beverages. This structure remained unchanged in subsequent editions, facilitating the monitoring of beverage availability trends over time.
